# Essential Oil Coating: Mediterranean Culinary Plants as Grain Protectants against Larvae and Adults of *Tribolium castaneum* and *Trogoderma granarium*

**DOI:** 10.3390/insects13020165

**Published:** 2022-02-03

**Authors:** Nikos E. Papanikolaou, Nickolas G. Kavallieratos, Vassilios Iliopoulos, Epameinondas Evergetis, Anna Skourti, Erifili P. Nika, Serkos A. Haroutounian

**Affiliations:** 1 Laboratory of Agricultural Zoology and Entomology, Department of Crop Science, Agricultural University of Athens, 75 Iera Odos Str., 11855 Athens, Greece; annaskourti@aua.gr (A.S.); erifilinika@aua.gr (E.P.N.); 2 Laboratory of Nutritional Feeding, Department of Animal Science, School of Animal Biosciences, Agricultural University of Athens, 75 Iera Odos Str., 11855 Athens, Greece; heliopoylos@hotmail.com (V.I.); epaev@aua.gr (E.E.); sehar@aua.gr (S.A.H.)

**Keywords:** micromulsion, postharvest pest, grain coating, essential oil, stored-product pest

## Abstract

**Simple Summary:**

The protection of stored agricultural products has been established as a global priority serving both food safety and security. Toxicity and residual issues of synthetic insecticides shifted the research focus towards natural pest control agents. In this context, six edible plants were selected for the conduction of a novel bioprospecting effort aiming to identify potential control agents against the red flour beetle, *Tribolium castaneum* (Herbst) (Coleoptera: Tenebrionidae) and the khapra beetle, *Trogoderma granarium* Everts (Coleoptera: Dermestidae). The proposed bioprospecting effort aims to identify the chemodiversity of essential oils (EOs) and exploit the potential of EO-based microemulsion (ME) coating as alternative tools for the management of the tested stored-product insects and the concomitant postharvest losses. Elevated toxicity was recorded against *T. castaneum* larvae and *T. granarium* adults. The fact that these EO-based MEs originate from culinary plants renders them safe for human consumption. The present study pioneers the utilization of EO-based MEs as grain protectants in the form of grain coating.

**Abstract:**

Postharvest agricultural losses constitute a major food security risk. In contrast, postharvest protection is strongly linked with food safety. The present study aims to develop novel postharvest protection tools through a bioprospecting protocol utilizing edible essential oils (EOs) as grain coatings. For this purpose, six Mediterranean culinary plants were selected for evaluation. The EOs of juniper, *Juniperus phoenicea* L. (Pinales: Cupressaceae), marjoram, *Origanum majorana* L. (Lamiales: Lamiaceae), oregano, *Origanum vulgare* ssp. *hirtum* (Link) A.Terracc. (Lamiales: Lamiaceae), bay laurel, *Laurus nobilis* L. (Laurales: Lauraceae) and tarhan, *Echinophora tenuifolia* ssp. *sibthorpiana* (Guss.) Tutin (Apiales: Apiaceae) were retrieved through steam distillation, while lemon, *Citrus limon* (L.) Osbeck (Sapindales: Rutaceae) EO was retrieved through cold press extraction. All EOs were formulated to microemulsions (MEs) and applied uniformly as a coating on wheat against larvae and adults of *Tribolium castaneum* (Herbst) (Coleoptera: Tenebrionidae) and *Trogoderma granarium* Everts (Coleoptera: Dermestidae). All EO-based MEs have been evaluated for the first time as grain coatings. They caused moderate to high mortality to *T. castaneum* larvae (67.8–93.3% 14 days post-exposure) and *T. granarium* adults (70.0–87.8% after 7 days of exposure). *Citrus limon*, *O. majorana* and *E. tenuifolia* ssp. *sibthorpiana* EO-based MEs were the most efficient against *T. castaneum* larvae, by exhibiting 93.3%, 91.1% and 90.0% mortality 14 days post-exposure, respectively. *Origanum majorana*, *L. nobilis* and *J. phoenicea* EO-based MEs were the most efficient against *T. granarium* adults, exhibiting 87.8%, 84.4% and 83.3% mortality after 7 days of exposure, respectively. These results indicate that EO-based ME coating is a potent tool against the tested postharvest pests.

## 1. Introduction

Food security, under the perspectives of global population increase and shifting consumer habits, is one of the main future challenges for the agricultural sector [1,2,3]. In the European Union, the main instrument of agricultural development, i.e., the Common Agricultural Policy (CAP), has set as a target, in order to address this challenge, the increase of agricultural production by 20% in 2030 [4]. The Food and Agricultural Organization (FAO) provides an alternative perspective of food security by focusing on the postharvest losses of agricultural production which is estimated to be 10% in developed countries and exceed 20% in developing countries [5,6].

The interconnectivity of these two approaches is well established and has been highlighted as a challenge since the Bronze Age [7,8,9]. While postharvest pest infection is horizontal across the sum of agricultural products [10], it is elevated as a significant risk for global food security in the case of staple food infections, with cereals and legumes being prominent among them [11]. Numerous efforts towards the eradication of fungal infestations have been summarized by Schmidt et al. [12], concluding the necessity of a combinatorial approach against the microbe contamination of stored grains such as cold atmospheric pressure plasma and electrolyzed water treatments. Similar advances may also be traced for insect and mite postharvest pests, focusing on their judicious management [13]. A prominent position among orders of insects that are related to stored products is reserved for Coleoptera. More than 600 coleopteran species have been identified as pests of food commodities with a cosmopolitan anthropochore distribution and have been established as major factors of stored-grain degradation [14]. Adults and larvae of these holometabolous insects cause serious direct and indirect damages in stored products by biting and chewing with their mandibles. Although some adults of these species do not feed or rarely feed upon stored commodities, they are also important since they are the vehicles of reproduction. Furthermore, due to the fact that several coleopterans are strong fliers, they can be easily distributed within/among storage facilities and between field and storage facilities [5,14].

Despite the fact that chemical insecticides are effective against a wide spectrum of insects, they may negatively affect the environment and health of consumers [15,16]. It is well documented that stored-product insects have developed resistance to major classes of insecticides, such as pyrethroids and organophosphates, due to their continuous exposure to synthetic insecticides [17,18,19,20]. Therefore, recent advances in policies but also on the regulation of active substances emphasize the use of non-synthetic plant protection products [21,22]. While novel approaches such as cold plasma [23] and ozone [24] treatments have been proven efficient, they have also presented significant side effects, mostly in relation to the nutritional value and physical and chemical properties of grains. On the other hand, natural products have been demonstrated as a promising source of plant protection tools [22,25,26,27].

Among natural products, essential oils (EOs) constitute a distinct class, representing complex clusters of plant secondary metabolites, with decreased mammalian toxicity and ecosystem penetrability and a selective mode of action circumnavigating the risk of resistance development [28,29]. Essential oils have been studied in relation to their fumigant toxicity [30,31,32] and their contact toxicity [33,34,35], but only recently has there been a focus on novel application methods of EOs [36,37]. This research interest became fruitful by providing a solid methodological approach for the application of volatile compounds as stored grain coatings in the form of nanoemulsions (NE) [38,39,40,41]. Microemulsions (ME), on the other hand, are kinetically stable, oily droplets in water, with a Surfactant-to-Oil Ratio (SOR) usually higher than 2 [42]. Previous reports have indicated that MEs are effective against different species of insects [43,44].

The present study builds upon the advances of MEs and aims at ameliorating the knowledge on EOs toxicity against stored-product pests through the introduction of a novel bioprospecting protocol. For this purpose, EO-based MEs have been implemented for first time as grain coating agents. The subjects of investigation were retrieved from the Greek biodiversity pool with a distinct focus on edible and/or culinary plants [45,46,47,48,49,50]. This way, the EO-based ME grain coating will be compatible with human consumption. The ME preparation utilized food grade emulsifiers and solvents. As target pests, the red flour beetle, *Tribolium castaneum* (Herbst) (Coleoptera: Tenebrionidae), a highly destructive stored-product insect pest of Indo-Australian origin [51,52], and the khapra beetle, *Trogoderma granarium* Everts (Coleoptera: Dermestidae), a highly destructive pest affecting a wide variety of commodities worldwide of animal and plant origin [52,53,54,55,56,57] and included in the 100 most important invasive species worldwide [58], were selected.

## 2. Materials and Methods

### 2.1. Plant Material

Plant material from six Greek indigenous culinary species was examined in the present study. These species are lemon, *Citrus limon* (L.) Osbeck (Sapindales: Rutaceae), juniper, *Juniperus phoenicea* L. (Pinales: Cupressaceae), bay laurel, *Laurus nobilis* L. (Laurales: Lauraceae), tarhan, *Echinophora tenuifolia* ssp. *sibthorpiana* (Guss.) Tutin (Apiales: Apiaceae), marjoram, *Origanum majorana* L. (Lamiales: Lamiaceae) and oregano, *Origanum vulgare* ssp. *hirtum* (Link) A.Terracc. (Lamiales: Lamiaceae) (Table 1). All authentic samples utilized for the identification of EO compounds were obtained from Sigma-Aldrich (Steinheim, Germany), except for germacrene D and *α*-thujene, which had been isolated in the context of previous studies. The food grade emulsifier, TWEEN^®^ 20 (97%) (Sigma-Aldrich, Steinheim, Germany), was utilized for the preparation of formulations.

### 2.2. Commodity

Hard wheat, *Triticum durum* Desf. (var. Claudio), commercially acquired, that was free from pest infestations and pesticides was used in the bioassays. Wheat was sieved to remove the impurities and stored at subzero temperatures for several months. Prior to experimentation, the wheat was warmed under room temperature. The moisture content was 12.2% as determined by a calibrated moisture meter (mini GAC plus, Dickey-John Europe S.A.S., Colombes, France).

### 2.3. Insect Species

The insect species used in the bioassays were obtained from cultures that are kept at the Laboratory of Agricultural Zoology and Entomology, Agricultural University of Athens. The founding individuals of *T. castaneum* and *T. granarium* have been collected from Greek storage facilities since 2003 and 2014, respectively. The selected insect individuals of both species and developmental stages, as well as the conditions they were cultured in, were adapted from previous studies [40,59].

### 2.4. Essential Oil Isolation and Analysis

All EOs were obtained by hydro-distillation using a modified Clevenger apparatus, according to previously described procedure [60]. The isolation yields of all EOs are included in Table 1. The chemical composition of EOs was determined on a gas chromatographer (GC) coupled to a mass spectrometer (MS) and Flame Ionization Detector (FID) in accordance with a previously described method [60]. Mass spectra were compared with NIST 11 and Willey 275 databases and authentic samples where available.

### 2.5. Bioassays

A stock solution of EO and TWEEN^®^ 20 (Sigma-Aldrich Chemie GmbH, Taufkirchen, Germany) (1:1) was prepared from each plant according to the specifications presented in Table 1. The analogy of the EO and the emulsifier was decided according to previously a described protocol [61] in order to produce an ME upon dilution with water. The EO-based ME stock solutions were tested at the concentration of 1000 ppm, where water (0.05% TWEEN^®^ 20) served as control. The selection of this concentration was based on preliminary tests. The experiments were conducted according to Kavallieratos et al. [62], while the application protocol of the MEs followed the guidelines provided by Golden et al. [38]. In this task, treatments were performed on plates, each one representing a treatment replicate. Quantities of 0.20 kg of wheat were each sprayed with 1 mL of the test solution by using an AG-4 airbrush (Mecafer S.A., Valence, France). Different plates were used per spraying. Between treatments, the airbrush was cleaned with alcohol to avoid cross contamination. The sprayed whole wheat was inserted separately in 1 kg plastic canisters and was shaken for 10 min to achieve the balanced distribution of the EO-based MEs on the whole quantity of grains. Three subsamples of 10 g were obtained and placed in Petri dishes (9 cm diameter, 1.5 cm height) using a different scoop that was inside each canister. The covers of the dishes bore a circular opening (1.5 cm diameter) on their centers that was covered by muslin cloth. Thus, the content of the dishes would be adequately aerated. The upper internal vertical sides of each dish were covered by polytetrafluoroethylen (60 wt % dispersion in water) (Sigma-Aldrich Chemie GmbH, Taufkirchen, Germany) to prevent the escape of insects from the treated wheat. The samples of 10 g of wheat were weighed with a Precisa XB3200D electronic balance (Alpha Analytical Instruments, Gerakas, Greece) on filter paper. Paper was changed each time weighing was conducted. After this, 10 adults or larvae of each species were transferred. The mortality of larvae of both species and *T. castaneum* adults was determined after 1, 3, 7 and 14 days of exposure, while the mortality of *T. granarium* adults was determined after 1, 3 and 7 days due to the shorter adult longevity of this species [63].

### 2.6. Data Analysis

Mortality in the control treatments was low (<5%) for both species, therefore no correction was considered necessary for the mortality values (correction is conducted when control mortality ranges between 5% and 20% [64]). In order to normalize the variance, mortality data were log (x + 1) transformed prior to being submitted to ANOVA separately for each tested species and life stage [62,65]. The pairwise comparisons were conducted by using the Fischer LSD test (α = 0.05). All the analyses were performed with SigmaPlot 14.0 [66].

## 3. Results

### 3.1. Phytochemical Analysis

**Figure 1:**
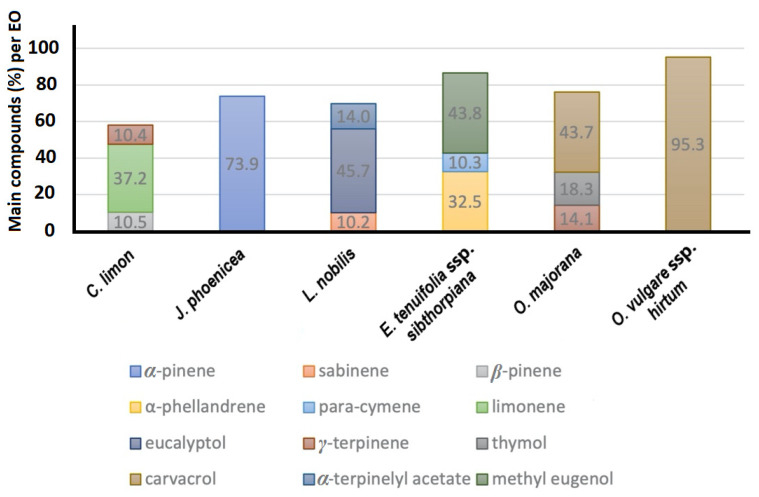
Main compounds of the EOs (>10%).

The results of the EO analysis revealed the presence of 48 phytochemical compounds, which are explicitly presented in Table 2. The main compounds in each plant’s EO are presented in Figure 1, while the presence of principal and secondary molecular structures in each EO are included in Table 1. From the EOs included in the present study, *O. vulgare* ssp. *hirtum* and *O. majorana* presented phenol carvacrol as the main compound, while in *O. majorana*, isomer thymol was also present in comparable quantity. *Citrus limon* and *J. phoenicea* EOs contain limonene and *α*-pinene as major compounds, respectively. *Laurus nobilis* EO was found to contain eucalyptol as a major compound, and *E. tenuifolia* ssp. *sibthorpiana* almost equal amounts of methyl eugenol and α-phellandrene.

### 3.2. Insecticidal Activity against T. castaneum

The mean mortality rate of *T. castaneum* larvae was significantly increased 1, 3 and 7 days after application of the EO-based MEs (Table 3). Thereafter, a significant increase in larval mortality was detected only for the application of the *C. limon* EO-based ME. Mean mortality rates of *T. castaneum* larvae 14 days after the application of *C. limon*, *J. phoenicea*, *L. nobilis*, *E. tenuifolia* ssp. *sibthorpiana*, *O. majorana* and *O. vulgare* ssp. *hirtum* EO-based MEs were 93.3%, 67.8%, 77.8%, 90.0%, 91.1% and 87.8%, respectively. However, all the tested EO-based MEs showed low mortality on *T. castaneum* adults. Thus, the observed mean mortality rates were 16.7%, 26.7%, 34.4%, 17.8%, 24.4% and 25.6% on wheat treated with *C. limon*, *J. phoenicea*, *L. nobilis*, *E. tenuifolia* ssp. *sibthorpiana*, *O. majorana* and *O. vulgare* ssp. *hirtum* EO-based MEs, respectively, 14 days post-exposure.

### 3.3. Insecticidal Activity against T. granarium

Concerning the efficacy on *T. granarium*, the tested EO-based MEs showed low mortality on insects’ larvae (Table 4). Depending on plant species, the efficacy of EO-based MEs ranged from 8.9% (*L. nobilis*) to 30.0% (*O. majorana*) 14 days after the exposure. However, *T. granarium* adults showed an increasing mortality rate after 1, 3 and 7 days exposure. Thus, mean mortality rates of insects’ adults 7 days after the application of *C. limon*, *J. phoenicea*, *L. nobilis*, *E. tenuifolia* ssp. *sibthorpiana*, *O. majorana* and *O. vulgare* ssp. *hirtum* EO-based MEs on wheat were 72.2%, 83.3%, 84.4%, 70.0%, 87.8% and 82.2 %, respectively.

## 4. Discussion

The composition of *O. majorana* EO is compatible with previous reports that indicate both thymol [67] and carvacrol [68] as main compounds and its significant chemical diversity is recognized. It must be noted that previous analyses of *O. majorana* EO from Greece [69] have also revealed the molecule of cymene as a major compound but not γ-terpinene. The *L. nobilis* EO composition is also consistent with previous reports identifying eucalyptol as the main compound [70], while the major compound α-terpinenyl acetate has also been reported [71]. The composition of *E. tenuifolia* ssp. *sibthorpiana*, *O. vulgare*, *C. limon* and *J. phoenicea* EOs has been presented and extensively discussed in previous studies [60,72,73].

EOs exhibit a significant range of pesticidal activities [32,72,74,75,76]. They can be produced easily, in a green and low-cost way, i.e., not including organic solvents or complicated methods of extraction [22]. In addition, EOs provide secondary metabolites that can act as modifying agents to resistant organisms, by inhibiting their proteins [77]. *Citrus limon* EO has been previously studied as a fumigant against *T. castaneum* adults with elevated efficacy [78,79]. By testing the contact toxicity and repellency of *O. majorana* EO against *T. castaneum* adults, Teke et al. [80] found potent repellency (97.2%) but not insecticidal activity after 3 days of exposure. Likewise, *O. vulgare* EO exhibited high fumigant and repellent properties against *T. castaneum* adults [81,82]. The evaluation of *L. nobilis* EO against *T. castaneum* in semolina suggested significant insecticidal potentials with simultaneous retention of crucial semolina quality characteristics [83].

Our study clearly shows the effectiveness of the EO-based MEs of the Mediterranean plants *C. limon, J. phoenicea, L nobilis, E. tenuifolia ssp. sibthorpiana, O. majorana* and *O. vulgare* ssp. *hirtum* against the two tested stored-product insect pests. *Citrus limon*, *O. majorana* and *E. tenuifolia* ssp. *sibthorpiana* EO-based MEs were the most effective for the management of *T. castaneum* larvae, by killing 93.3%, 91.1% and 90.0% of the exposed individuals after 14 days of exposure, respectively. *Origanum majorana*, *L. nobilis* and *J. phoenicea* EO-based MEs killed 87.8%, 84.4% and 83.3% of *T. granarium* adults after 7 days of exposure, respectively. The findings indicate that the evaluated EO-based MEs are effective grain protectants for the management of *T. granarium* adults and *T. castaneum* larvae. So far, limited research has been conducted on EOs as grain protectants. For example, Demirel et al. [84] suggested that the EOs extracted from rosemary, *Rosmarinus officinalis* L. (Lamiales: Lamiaceae)*, O. majorana* and thyme, *Thymus vulgaris* L. (Lamiales: Lamiaceae), can be used as a potential source of environment-friendly wheat protectants for the control of the confused flour beetle, *Tribolium confusum* Jacquelin du Val (Coleoptera: Tenebrionidae). Recently, Kavallieratos et al. [85] showed that EOs obtained from horse mint, *Mentha longifolia* (L.) Huds. (Lamiales: Lamiaceae)*,* wormseed, *Dysphania ambrosioides* (L.) Mosyakin & Clemants (Caryophyllales: Chenopodioideae), stemless carline thistle, *Carlina acaulis* L. (Asterales: Compositae), and anise, *Pimpinella anisum* L. (Apiales: Apiaceae) are stored maize and wheat protectants against two stored-product insects pest, the larger grain borer, *Prostephanus truncatus* (Horn) (Coleoptera: Bostrychidae) and *T. granarium*. In addition, Pavela et al. [76] revealed that the essential oils of *Ferula assa-foetida* L. (Apiales: Apiaceae) and *Ferula gummosa* Boiss. (Apiales: Apiaceae) were highly effective against adults of *T. granarium* when applied on stored wheat.

The insect developmental stage is a critical aspect of the efficacy of the EO as grain protectants [62,76]. *Tribolium castaneum* larvae were more susceptible to the EO-based MEs than adults. On the basis of our results, *C. limon, J. phoenicea, L. nobilis, E. tenuifolia* ssp. *sibthorpiana, O. majorana* and *O. vulgare* ssp. *hirtum* EO-based MEs provided low adult mortality levels, ranging from 16.7% to 34.4% 14 days post-exposure. In contrast, in the case of larvae, the same EO-based MEs provided moderate to high mortality levels, ranging from 67.8% to 93.3% after 14 days of exposure. Similarly, a 6% (w/w) *Hazomalania voyronii* (Jum.) Capuron (Laurales: Hernandiaceae) EO-based NE caused low mortality to *T. castaneum* adults (i.e., 18.7%) vs. high mortality to larvae (i.e., 97.4%) 7 days post-exposure [40]. However, in the case of *T. granarium*, adults were more vulnerable than larvae. The tested EO-based MEs caused the death of a low percentage of the exposed *T. granarium* larvae (8.9–30.0%) after 14 days of exposure, while they caused elevated mortality (72.2–87.7%) to *T. granarium* adults, 7 days post-exposure. It is well documented that *T. granarium* larvae are more tolerant than adults to EOs [85] and compounds of botanical origin [22,40,62] when applied on wheat. This could be attributed to the long and dense hairs that cover the body of larvae, protecting them from coming in contact with the treated wheat [86]. In contrast, larvae of *Tribolium* spp. are covered by few hairs [87], an issue that increases the likelihood of their contact with the toxicant. The increased tolerance of *T. castaneum* adults in comparison to larvae could be attributed to the different structure of their cuticles [88]. Another hypothesis is that the expression of the *TcCYP6BQ7* gene, which is responsible for the detoxification of plant toxicants, is higher in adults than larvae of *T. castaneum* [89].

In general, pesticide treatment with synthetic insecticides is a common practice against stored-product insect pests [36,62]. However, food safety is generally associated with integrated pest management, aiming to use alternative protectants and/or low-risk pesticides [22,90,91,92]. Botanicals are low-risk alternative products, linked with reduced regulatory registration procedures [93]. Our results lean towards this direction, as we showed that the EO-based MEs of several plants have the potential to serve as efficient tools against major stored-product insect pests. Developing grain protectants from plants will bring benefits to the food supply chain with simple and cost-effective products of insecticidal activity [94].

## 5. Conclusions

All EO-based MEs included in the current bioprospecting study exhibit the prevailing phytochemical EO profile for the respective plant taxa. This fact enhances the replicability and upscale of the findings, since the exploited raw materials are widely available in nature [34,45,46,47,48,49,50,95,96]. In addition, we expect our results to have bearing on the control and the integrated pest management of stored-product insect pests. Further research on the insecticidal activity of several Mediterranean plants as grain protectants will gather together more information towards efficient, more sustainable management strategies in storage facilities.

## Figures and Tables

**Table 1:** Essentials oils, plant origin and stock solution composition.

Taxon	Source	Stock Solution		
		EO	TWEEN^®^ 20	Water
*Citrus limon*	Industrial byproduct	20%	20%	60%
*Juniperus phoenicea*	Wild gathered	20%	20%	60%
*Laurus nobilis*	Cultivated	20%	20%	60%
*Echinophora tenuifolia* ssp. *sibthorpiana*	Wild gathered	20%	20%	60%
*Origanum majorana*	Cultivated	20%	20%	60%
*Origanum vulgare* ssp. *hirtum*	Cultivated	20%	20%	60%

**Table 2:** EOs qualitative and quantitative composition. RI = Retention Index; Identification Method: a = MS, b = RI, c = comparison with authentic standards.

Compounds	RI	*C. limon*	*J. phoenicea*	*L. nobilis*	*E. tenuifolia* ssp. *sibthorpiana*	*O. majorana*	*O. vulgare* ssp. *hirtum*	Identification
*α*-thujene	930	0.6			0.2	1.5	0.1	a, b, c
*α*-pinene	939	2.3	73.9	3.9	0.6	0.9		a, b, c
camphene	954		0.5			0.3		a, b, c
sabinene	975		0.3	10.2	0.1			a, b, c
*β*-pinene	980	10.5	1.9	3.6	0.1	0.3		a, b
1-octen-3-ol	981					0.3		a, b
myrcene	991	2.0	3.3	0.9	0.2	1.5	0.2	a, b, c
α-phellandrene	1003		3.1	0.5	32.5	0.2		a, b
*α-terpinene*	1017			0.7	0.9	2.0	0.2	a, b
para-cymene	1025				10.3	0.1	1.0	a, b
ortho-Cymene	1027			0.8		8.1		a, b
limonene	1029	37.2						a, b, c
*β*-phellandrene	1031				6.5			a, b
eucalyptol	1032			45.7		t		a, b
*trans-β*-ocimene	1051	0.2						a, b
*γ*-terpinene	1060	10.4	0.2	0.7	0.6	14.1	0.9	a, b, c
*α*-terpinolene	1089	0.7	0.7		0.5		0.4	a, b
linalool	1098			1.7		0.2		a, b
nonanal	1101	0.2						a, b
camphor	1145		0.5					a, b
citronelal	1153	0.3						a, b
borneol	1168					0.5	0.2	a, b
4-terpineol	1178		0.2	2.4		0.5	0.1	a, b
*α*-terpineol	1189	0.3	0.4	3.0				a, b
neral	1238	1.2						a, b
carvacrol methyl ether	1245					0.7		a, b
piperitone	1253		0.1					a, b
bornyl acetate	1287			0.9				a, b
lavandulyl acetate	1290	0.9						a, b
thymol	1293				0.1	18.3	0.6	a, b
carvacrol	1299				0.5	43.7	95.3	a, b, c
citral	1320	2.0						a, b
*δ*-eIemene	1338		0.1					a, b
*a*-terpinelyl acetate	1351		1.1	14.0				a, b
eugenol	1359			2.5				a, b
neryl acetate	1362	1.2						a, b
*β*-elemene	1391		0.2					a, b
methyl eugenol	1406			1.2	43.8			a, b
*β*-caryophyllene	1419	0.6	1.3			1.9	0.4	a, b, c
*α*-bergamotene	1435		1.0					a, b
*γ*-elemene	1437		0.2					a, b
*α*-humulene	1455		0.6					a, b
germacrene D	1485		4.2					a, b, c
valencene	1496	0.2						a, b
bicyclogermacrene	1500	0.1						a, b
*β*-bisabolene	1506	1.5				0.3	0.2	a, b
*δ*-cadinene	1523		0.2					a, b
germacrene B	1561		1.2					a, b
Total		72.4	95.3	92.7	96.8	95.4	99.8	

**Table 3:** Mean mortality rate (% ± SE) of *T. castaneum* (larvae and adults) 1, 3, 7 and 14 days after application with EO-based MEs. Means in the same row followed by different uppercase letters are significantly different; means in a column followed by different lowercase letters are significantly different (Fisher LSD test, α = 0.05).

Plant Species	Developmental Stage	Days after the Treatment				DF	*F*	*p*
		1 Day	3 Days	7 Days	14 Days			
*C. limon*	Larvae	8.9 ± 2.0 Aa	40.0 ± 7.1 Bab	72.2 ± 6.0 Cad	93.3 ± 2.4 Da	3	59.772	<0.001
	Adults	0.0 ± 0.0 Aa	0.0 ± 0.0 Aa	3.3 ± 2.4 Aa	16.7 ± 6.9 Ba	3	5.095	0.005
*J. phoenicea*	Larvae	13.3 ± 5.3 Aa	32.2 ± 6.8 Bab	54.4 ± 8.2 Cbc	67.8 ± 8.5 Cb	3	11.801	<0.001
	Adults	2.2 ± 1.5 Aa	7.78 ± 4.0 Aa	13.3 ± 5.3 ABa	26.7 ± 6.5 Ba	3	4.960	0.006
*L. nobilis*	Larvae	14.4 ± 4.1 Aa	33.3 ± 5.3 Bab	57.8 ± 5.7 Ccd	77.8 ± 8.0 Cbcd	3	22.470	<0.001
	Adults	5.6 ± 4.4 Aa	8.9 ± 5.6 Aa	15.6 ± 5.8 Aa	34.4 ± 6.0 Ba	3	5.555	0.003
*E. tenuifolia* ssp. *sibthorpiana*	Larvae	13.3 ± 4.4 Aa	40.0 ± 4.1 Bbc	77.8 ± 2.2 Ca	90.0 ± 2.9 Cad	3	83.773	<0.001
	Adults	0.0 ± 0.0 Aa	2.2 ± 1.5 Aa	5.6 ± 1.8 Aa	17.8 ± 3.6 Ba	3	13.814	<0.001
*O. majorana*	Larvae	15.6 ± 1.8 Aa	57.8 ± 5.5 Bc	80.0 ± 4.7 Ca	91.1 ± 3.5 Cac	3	80.600	<0.001
	Adults	7.8 ± 4.7 Aa	14.4 ± 5.3 Aa	17.8 ± 7.2 Aa	24.4 ± 6.5 Aa	3	1.386	0.265
*O. vulgare* ssp. *hirtum*	Larvae	11.1 ± 4.2 Aa	44.4 ± 6.5 Bbc	72.2 ± 4.9 Cad	87.8 ± 3.2 Cad	3	50.233	<0.001
	Adults	0.0 ± 0.0 Aa	2.2 ± 1.5 Aa	4.4 ± 1.8 Aa	25.6 ± 4.8 Ba	3	22.016	<0.001
DF	Larvae	5	5	5	5			
	Adults	5	5	5	5			
*F*	Larvae	0.411	2.409	3.796	3.431			
	Adults	1.605	2.296	1.844	1.232			
*p*	Larvae	0.839	0.050	0.005	0.010			
	Adults	0.177	0.060	0.122	0.309			

**Table 4:** Mean mortality rate (% *±* SE) of *T. granarium* (larvae and adults) 1, 3, 7 and 14 days after application with EO-based MEs. Means in the same row followed by different uppercase letters are significantly different; means in a column followed by different lowercase letters are significantly different (Fisher LSD test, α = 0.05).

Plant Species	Developmental Stage	Days after the Treatment				DF	*F*	*p*
		1 Day	3 Days	7 Days	14 Days			
*C. limon*	Larvae	3.3 ± 1.7 Aa	4.4 ± 2.4 Aab	10.0 ± 4.1 Aac	13.3 ± 3.7 Aab	3	2.282	0.098
	Adults	8.9 ± 2.6 Aa	26.7 ± 2.4 Ba	72.2 ± 4.0 Ca	N/A	2	111.285	<0.001
*J. phoenicea*	Larvae	3.3 ± 1.7 Aa	16.7 ± 2.3 Bc	26.7 ± 4.4 BCb	34.4 ± 4.4 Cc	3	16.049	<0.001
	Adults	14.4 ± 2.9 Aa	27.8 ± 2.8 Ba	83.3 ± 5.3 Ca	N/A	2	90.937	<0.001
*L. nobilis*	Larvae	3.3 ± 1.7 Aa	4.4 ± 1.8 Aab	8.9 ± 2.6 Aac	8.9 ± 2.6 Aa	3	1.719	0.183
	Adults	11.1 ± 2.0 Aa	25.6 ± 2.4 Ba	84.4 ± 2.9 Ca	N/A	2	214.397	<0.001
*E. tenuifolia* ssp. *sibthorpiana*	Larvae	1.1 ± 1.1 Aa	8.9 ± 2.6 Bbc	16.7 ± 3.7 BCbc	20.0 ± 3.3 Cbd	3	9.122	<0.001
	Adults	8.9 ± 2.6 Aa	17.8 ± 2.8 Aa	70.0 ± 7.5 Ba	N/A	2	52.800	<0.001
*O. majorana*	Larvae	2.2 ± 1.5 Aa	16.7 ± 4.4 Bc	23.3 ± 6.0 BCbc	30.0 ± 6.0 Ccd	3	6.232	0.002
	Adults	6.7 ± 3.3 Aa	21.1 ± 3.5 Ba	87.8 ± 5.7 Ca	N/A	2	90.808	<0.001
*O. vulgare* ssp. *hirtum*	Larvae	2.2 ± 1.5 Aa	5.6 ± 2.4 Aab	12.2 ± 1.5 BCac	14.4 ± 1.8 Cab	3	10.171	<0.001
	Adults	12.2 ± 2.2 Aa	23.3 ± 1.7 Ba	82.2 ± 1.5 Ca	N/A	2	310.817	<0.001
DF	Larvae	5	5	5	5			
	Adults	5	5	5	N/A			
*F*	Larvae	0.356	4.143	3.310	7			
	Adults	1.142	2.040	2.157	N/A			
*p*	Larvae	0.876	0.003	0.012	<0.001			
	Adults	0.351	0.090	0.075	N/A			

## Data Availability

Data are contained within the article.
